# Acupuncture and chiropractic care for chronic pain in an integrated health plan: a mixed methods study

**DOI:** 10.1186/1472-6882-11-118

**Published:** 2011-11-25

**Authors:** Lynn L DeBar, Charles Elder, Cheryl Ritenbaugh, Mikel Aickin, Rick Deyo, Richard Meenan, John Dickerson, Jennifer A Webster, Bobbi Jo Yarborough

**Affiliations:** 1 Kaiser Permanente, The Center for Health Research, Portland, Oregon, USA; 2 Department of Family & Community Medicine, University of Arizona, Tucson, Arizona, USA; 3 Department of Family Medicine, Oregon Health and Science University, Portland, Oregon, USA

## Abstract

**Background:**

Substantial recent research examines the efficacy of many types of complementary and alternative (CAM) therapies. However, outcomes associated with the "real-world" use of CAM has been largely overlooked, despite calls for CAM therapies to be studied in the manner in which they are practiced. Americans seek CAM treatments far more often for chronic musculoskeletal pain (CMP) than for any other condition. Among CAM treatments for CMP, acupuncture and chiropractic (A/C) care are among those with the highest acceptance by physician groups and the best evidence to support their use. Further, recent alarming increases in delivery of opioid treatment and surgical interventions for chronic pain--despite their high costs, potential adverse effects, and modest efficacy--suggests the need to evaluate real world outcomes associated with promising non-pharmacological/non-surgical CAM treatments for CMP, which are often well accepted by patients and increasingly used in the community.

**Methods/Design:**

This multi-phase, mixed methods study will: (1) conduct a retrospective study using information from electronic medical records (EMRs) of a large HMO to identify unique clusters of patients with CMP (e.g., those with differing demographics, histories of pain condition, use of allopathic and CAM health services, and comorbidity profiles) that may be associated with different propensities for A/C utilization and/or differential outcomes associated with such care; (2) use qualitative interviews to explore allopathic providers' recommendations for A/C and patients' decisions to pursue and retain CAM care; and (3) prospectively evaluate health services/costs and broader clinical and functional outcomes associated with the receipt of A/C relative to carefully matched comparison participants receiving traditional CMP services. Sensitivity analyses will compare methods relying solely on EMR-derived data versus analyses supplementing EMR data with conventionally collected patient and clinician data.

**Discussion:**

Successful completion of these aggregate aims will provide an evaluation of outcomes associated with the real-world use of A/C services. The trio of retrospective, qualitative, and prospective study will also provide a clearer understanding of the decision-making processes behind the use of A/C for CMP and a transportable methodology that can be applied to other health care settings, CAM treatments, and clinical populations.

**Trial registration:**

ClinicalTrials.gov: NCT01345409

## Background

We describe here our study designed to understand clinically meaningful outcomes (from both patient and provider perspectives) of acupuncture and chiropractic (A/C) care as delivered in routine practice settings for the treatment of chronic musculoskeletal pain (CMP). The centerpiece of the study is a prospective cohort study. However, before we undertake this phase of work, we will conduct an analysis of electronic medical record (EMR) data and qualitative data collection to provide the foundation for identifying a more meaningful comparison of outcomes between those receiving and not receiving A/C care. Our goal is to test an exportable methodological approach that can be used to understand critical outcomes associated with the receipt of an array of treatment services in everyday practice settings for a realistically diverse set of patients.

Chronic pain is a highly prevalent condition, often resulting in large decrements in health-related quality of life (QOL) and functional status, with substantial associated medical care costs, disability, and productivity loss [1-3]. CMP in particular is both prevalent and costly [4-7], affecting 60-80% of American adults at some point during their lives; CMP symptoms are among the top five reasons that patients visit clinics and emergency departments [6,8]. Recent alarming increases in delivery of opioid treatment and surgical interventions for chronic pain--despite their high costs, potential adverse effects, and modest efficacy [9-12]--suggest the need to evaluate outcomes associated with promising non-pharmacological/non-surgical approaches for CMP management and treatment, including complementary and alterative medicine (CAM). Americans seek CAM treatments far more often for CMP than for any other condition [13].

Substantial recent research has examined the biological basis and efficacy of many types of CAM therapies but, despite calls for effectiveness-oriented research, "real-world" use of CAM remains understudied [14,15]. Use of CAM for CMP appears to be increasing. A national survey [13] found that 38% of U.S. adults used some form of CAM, most commonly for relief of back and neck pain, joint pain and stiffness, arthritis, and other musculoskeletal conditions. Among CAM treatments for CMP, acupuncture and chiropractic care are considered the most highly accepted by physician groups [16,17] with the best evidence to support their use [18-21]. Nearly 90% of states mandate insurance reimbursement for chiropractic care and approximately 25% do so for acupuncture [22]. Further, a survey of acupuncturists and chiropractors in Massachusetts, Arizona, and Washington found that back pain was the most common reason given by patients for seeking treatments; overall, 40-76% of patients included CMP among their reasons for seeking such treatment [23].

Patients also report high levels of satisfaction with acupuncture [24] and chiropractic care [18]. A Consumer Reports survey found that while more than half of the respondents reported being highly satisfied with care from acupuncturists (53%) and chiropractors (59%) for back pain, only 44% reported similar satisfaction with care from specialist physicians and 34% with care from primary care physicians [25]. Despite some positive findings among observational studies and randomized controlled trials (RCTs) regarding the impact of acupuncture [18,26,27] and chiropractic care [18,28-30] on CMP, highly controlled trials have suggested that expectation and non-specific effects may be substantial contributors to observed treatment effects [31-33]. These findings highlight the importance of examining patient expectations and treatment decision-making factors when evaluating such outcomes. Given both the popularity of A/C for CMP treatment and outcome findings, an important next step is to examine the use of these CAM therapies for CMP as they are delivered by providers in routine practice settings.

**Figure 1:**
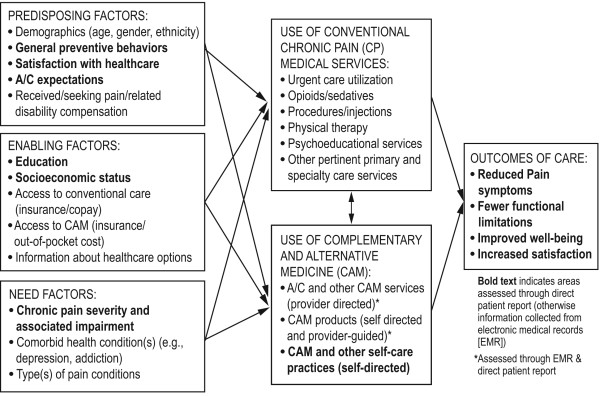
**Patient Factors Influencing the Decision to Use Acupuncture and/or Chiropractic Care (A/C) for Chronic Musculoskeletal Pain**.

Multiple recent reports [14,15,34,35] suggest the importance for health services research to explore models of organized health delivery that integrate CAM with conventional medicine. Until recently, patients were likely to make decisions about whether to use CAM services without input from allopathic providers [36,37], but today's patients are increasingly "co-managed" by conventional and CAM clinicians. Most patients report using CAM and conventional medicine together and want the opportunity to discuss CAM use with their primary care providers, be respected for their beliefs, and be guided on their use of such treatments [38-40]. Many CAM therapies are used to complement, rather than replace, conventional medicine; therefore, it is important to identify a model that can serve as a unified framework for the decision to use A/C within this context. Thus, we chose a framework to guide our exploration of patients' decisions to use A/C based on a well-accepted model for general health care decision-making and use [41,42], that has been expanded to consider integration of CAM services (see Figure 1)[43]. This model for CAM use includes commonly used self-directed practices and products as well as provider services that are the central focus of this study. Further, the model includes indicators that may "pull" a person toward A/C use (e.g., responsibility for preventive self-care) or "push" patients (e.g., dissatisfaction with conventional medicine)[44,45]. Research on conventional medicine use suggests that enabling factors (e.g., access, information about forms of care) and need (e.g., type and level of impairment) are the primary drivers of health care decisions [46-48], but their relative importance has not been explored for A/C service use.

This project will use data collected from a prepaid group practice model health maintenance organization (HMO) that offers A/C coverage for CMP treatment; in this setting, enabling factors of access (insurance coverage, co-pays, lower out-of-pocket costs) will likely mitigate the influence of economic factors [47,49,50]. Consequently, we will be able to explore more fully those non-financial predisposing and need factors that affect A/C decision making. This framework also helps guide us toward the most important domains for measurement.

Finally, there is increased demand for innovative study designs using data from routine practice settings to compare strengths and weaknesses of various medical interventions. Ideal settings are health care systems that use EMRs, provide insurance coverage, and document the use of provider-delivered CAM services, such as A/C care. Innovative research designs in these settings can provide information for health care providers and patients about which CAM and conventional treatments are likely to provide the best clinical, functional, and quality-of-life outcomes for everyday users in routine practice settings [51-53]. These designs will include a more diverse range of study subjects than would RCTs and allow longer follow-up, facilitating identification of groups that may uniquely benefit or encounter complications. These settings permit study designs that can examine the full array of treatments and associated outcomes that patients may encounter.

Despite these advantages, making causal inferences in observational studies is more challenging than in RCTs because of confounding by indication. That is, patients (and/or doctors) choose treatments using information that may not be evident to researchers. Patients who receive a given treatment in everyday practice may be dissimilar from their counterparts who do not, in which case treatment outcomes may be at least partially related to unmeasured pre-treatment differences rather than the treatment received. For pre-treatment differences where we have valid information, recent analytic techniques offer the promise of identifying patients with similar probability of receiving a particular treatment [54-58]. In the event that one of these patients receives the treatment and the other does not, clinical outcomes may be validly compared for such individuals. Using such approaches can complement what can be achieved with RCTs relatively quickly and efficiently. Such observational studies can highlight important domains for subsequent confirmatory RCTs or point to patterns of utilization and outcomes that are not predicted by existing RCTs.

The aims of the present study are two-fold. The first goal is to examine who (i.e., CMP patients with what characteristics and history of clinical care) will have improved outcomes from A/C care, and to identify the specific characteristics of such care (e.g., duration, comprehensiveness of employed A/C modalities). The second goal is to test an exportable methodological approach that can be used to examine clinically meaningful outcomes for patients: (1) in different settings, (2) using different types of CAM or conventional medical services, with (3) different health care conditions and characteristics.

## Methods/Design

### Study Overview

**Figure 2:**
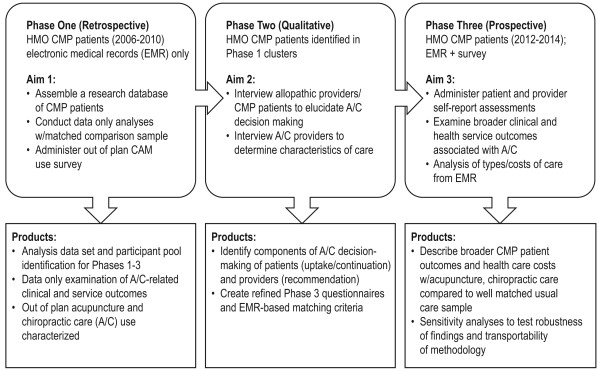
**Study Phases, Procedures, and Chronic Musculoskeletal Pain (CMP) Participant Flow**.

The overarching goal of this multi-phase, mixed methods study is to understand the real world implications for patient care and patient satisfaction of acupuncture and/or chiropractic treatment for CMP. Our study setting is a closed patient population HMO in which insurance coverage has been available for A/C treatments. In order to accomplish our goal, we divided our work into three complementary but distinct phases as summarized in Figure 2. Phase 1 involves assembling and exploring a research database with 5 years (2006-2010) of prospectively collected elements available within the HMO. We will identify HMO members who satisfy our study definition of CMP (see Table 1). The database will include members with evidence of using A/C services and a matched comparison group without such evidence. We will then compare these groups on clinical care outcomes and costs derived using EMR data. These analyses will be augmented with patient-reported out-of-plan A/C utilization. Phase 2 includes qualitative methods to describe the characteristics of A/C services received by HMO-based CMP patients and the decision-making processes of allopathic providers and patients in choosing such services; this will allow us to design the appropriate data collection tools and strategies for Phase 3. During Phase 3, we will conduct a longitudinal prospective cohort study of carefully matched samples of A/C and non-A/C patients. Participant selection will rely upon clustering/matching methods identified during Phase 1, refined by Phase 2 qualitative findings, and augmented by additional, direct-assessed patient-report baseline data. Outcomes will include EMR-derived utilization information and patient-reported outcomes. The patient-reported outcomes will cover clinical, psychosocial, quality of life, service utilization, and health care costs over 12 months. We will develop and/or refine these outcomes in the process of Phase 1 data exploration and Phase 2 qualitative research that explores outcomes that are clinically meaningful to conventional and CAM providers and are associated with high levels of patient satisfaction. Finally, we will compare outcomes from this augmented data set to those found during the Phase 1 EMR-only analyses, conducting sensitivity analyses to test the robustness of our findings and transportability of methodology. While phases 1 and 2 can stand alone, in this project they are preparatory to Phase 3, the longitudinal prospective cohort study.

**Table 1:** Overall Study Eligibility Criteria

***Inclusion criteria:***
≥ 18 years of age
Meets the following definition of musculoskeletal pain as determined review of the individual's electronic medical record (EMR) chart:
≥ 3 outpatient (emergency department, ambulatory visit, email and telephone encounters are acceptable) encounters spanning at least 180 days but no more than 18 months:
nonspecific chronic pain (nCP)
or chronic musculoskeletal pain (CMP). CMP includes arthritis, temporomandibular joint disorder, tension headaches, carpal tunnel syndrome, fibromyalgia and myofascial pain, pain in limb, pain in joint, back/neck pain, vertebral fractures, vertebral somatic dysfunction, spondylolysis, vertebral dislocation, spinal strains and sprains, spinal deformities
Three qualifying diagnoses can be achieved in any of the following ways:
Three CMP diagnoses
First diagnosis CMP and two subsequent diagnoses of nCP
First diagnosis CMP with one additional CMP diagnosis and one nCP
Diagnoses associated with a physical therapy visit will ***NOT ***be considered in identifying prospective participants
Only one CMP/nCP diagnosis can be counted in any given 7-day window to minimize the possibility that qualifying diagnoses reflect multiple visits/services for the same acute pain condition.
***Study participant exclusion criteria include:***
Medical history of malignant cancer other than non-melanoma skin cancer
Medical history of cognitive impairment (dementia, developmental delay, or psychosis)
Medical history of hospice or end-of-life palliative care

### Study Setting

This study is being conducted at Kaiser Permanente Northwest (KPNW), a large HMO in the Pacific Northwest with several features that create a uniquely favorable environment for this research. First, KPNW has an extensive history of CAM integration. Due to Washington State's 1998 law [59] mandating insurance coverage for a broad array of licensed providers (including chiropractors and acupuncturists), KPNW began to offer CAM coverage for all health plan members. Administration of this coverage is provided by a managed complementary care network that maintains electronic data from HCFA-1500 forms (including information on diagnoses, procedures, and dates of service) and these data are available to the project. Second, any health plan member can be referred by a KPNW clinician for A/C care for qualifying conditions, including acupuncture for CMP and chiropractic care for acute exacerbations of CMP conditions. Although some patients pay out-of-pocket for A/C care, our out-of-plan CAM use survey will help us discern this prevalence. Third, in 1995, KPNW implemented a comprehensive EMR system (HealthConnect) that has since captured A/C services and documented all outpatient provider encounters, diagnoses, referrals, laboratory and imaging studies, and prescriptions. The HMO provides most services in its own hospitals and clinics, yet data are compiled and available on reimbursable external services received by health plan patients. Together these provide an all-inclusive picture of members' covered health care utilization.

### Study participants

Study participants are health plan members aged 18 or older with evidence of CMP. To identify such patients from the EMR, we use ICD-9 codes consistent with CMP-related diagnoses, as outlined in Table 1. Musculoskeletal pain is thought to contribute to tension headaches, carpal tunnel syndrome, and temporomandibular disorders (TMD). Accordingly, and because A/C care is sometimes sought for the treatment of such conditions, we include participants with these diagnoses. To ensure chronicity of the pain disorder, visits must indicate such diagnoses or receipt of commensurate health services spanning at least 180 days. Exclusion criteria include history of cancer or cancer-related pain, hospice or other end-of-life palliative care, cognitive impairment (dementia, developmental delay, and psychosis) that would make it unlikely that the patient would receive A/C and/or could participate in study assessments. In later sections, we describe additional inclusion and exclusion criteria specific to the qualitative (Phase 2) and prospective cohort (Phase 3) portions of the study.

Preliminary analyses indicate that during a recent calendar year (2010), almost 24% (N = 71,584) of our 304, 034 current health plan members ≥ 18 years of age met criteria for current or recent CMP;, a high prevalence in the study setting even when using a stringent definition of chronic pain (at least six months' duration of symptoms). Further, 36% of these CMP patients met criteria for more than one type of chronic pain (see Table 2), suggesting the importance of including participants with more complex conditions (i.e., multiple simultaneous CMP ICD-9 diagnoses) to adequately examine care in routine settings.

**Table 2:** KPNW Health Plan Members with CMP in 2010

	No CAM referral in 2010	Chiropractic and/or acupuncture referral in 2010*
	Mean (SD) or % (N)	Mean (SD) or % (N)
Total members 18 and older with CMP	66768	4816
**Type of Chronic Pain**		
Back and Neck Pain	33248	4244
Joint Pain (including Osteoarthritis)	40308	2705
Fibromyalgia and other Myofascial Pain	7129	1125
Headache	7543	743
Two or more types of pain from CMP definition	34.3% (22904)	61.9% (2983)
Two or more types of pain from more general chronic pain definition	38.1% (25419)	63.4% (3052)
**Demographics**		
% female	61.4% (41058)	68.3% (3290)
Age	57.4 (15.5)	52.3 (14.8)
% White (1)	87.6% (54982)	87.4% (3996)
% Hispanic (2)	6.3% (2379)	6.8% (206)
**Other**		
Depression diagnosis	20.6% (13780)	26.8% (1292)
Sleep problems	0.46% (307)	0.89% (43)

*2224 chiropractic only, 2314 acupuncture only, 278 both.

(1) race information only available for 67, 319 health plan members, (2) ethnicity for 41, 098 health plan members

#### Phase 1: Construction of CMP sample and A/C Services Using EMR Data and an Out-of-Plan CAM Use Survey

The first phase of the project involves constructing a sample of HMO members with CMP over a five-year period (1/01/2006-12/31/2010). One difference between EMR-based and RCT-based research is the importance of the occurrence of medical events over time, in the absence of any research intervention. Each patient in the sample will have a virtual enrollment date, at which they first satisfied the CMP-eligibility criteria. All EMR data prior to that date correspond roughly to "baseline" data in an RCT, the primary difference being that the amount and duration of EMR-based events will almost certainly vary widely from patient to patient. As one moves forward from the virtual enrollment date, a given patient can be considered to be someone who uses A/C during that time period if they initiate A/C visits, or not if they rely solely on conventional care. Consequently, whether a patient is considered as treated (with A/C) or "control" (without A/C) depends on the time period over which outcome events are accumulated. This feature substantially complicates the analysis, above and beyond the absence of a formal intervention. Those who receive A/C treatment or are identified as "control" subjects can then be compared on clinical care outcomes and costs derived from EMR data.

Phase 1 includes a focus on analytic innovations that capitalize on our very large data set (122, 896 health plan members with EMR-identified CMP from 2006 to 2010) to describe pain-related services. We will also explore and compare outcomes found through multiple methods for identifying suitable comparable groups of patients (those who did and did not receive A/C services) and best controlling for potential confounders between the groups. This approach is consistent with recommendations for observational studies to employ multiple analytic strategies to ensure consistency of findings [60]. Reviewed below are our methods for organizing our data and creating descriptions of trajectories of pain care services (event-stream methods) followed by the three principal analytic approaches to be employed to match participants receiving A/C to suitable controls - selection models, propensity score analysis, and the analysis of matched comparison groups. These analytic methods will be oriented toward reduction of indication bias.

### Event-stream methods

Management of longitudinal patient records is difficult because patients receive services in diverse ways. Unlike clinical trials in which all measures are collected in the same time frame, clinical records include patients who visit the health plan at different times for different services, each displaying a unique, idiographic "trajectory of care." For CMP patients, for example, this might include use of patient education, physical therapy, and opiate medications over a particular period of time. The event-stream method allows a richer description of patient care over time, more consistent with how patients receive services in routine care. The method defines an efficient data interface between the clinical/administrative databases and the analysis datasets [61,62]. Event-stream methods can be used to construct the types of variables that can then be used in a conventional analysis. This approach simplifies both the data extraction and statistical analysis and allows a richer array of variables for identifying matches or clusters of patients.

### Selection models

To compare CMP patients who are users and non-users of A/C care on their health care utilization, we will need to adjust for features that might be associated with both the use of CAM (A/C care) and health care utilization overall. One of the challenges the project will address will be to determine the potential confounding factors from the EMR data alone. The chief threat to the validity of clinical observational studies is indication bias, in which factors that predict clinical outcomes are associated with treatment choices. This problem is not unique to biomedical research, and in fact the foundational development of selection models was carried out in economics [63]. Therefore, Heckman's selection models will be used to produce analyses based on joint estimation in a regression model (for the outcome) and a logistic regression model (for the treatment selection), which permits these two processes to be linked. Of all the approaches to be used in this project, this is the one that most closely mirrors statistical analysis in a conventional clinical trial.

### Propensity score analysis

Another method we will use to model the probability of being referred for and obtaining CAM treatment is propensity score analysis [56,59,64-66]. Propensity scoring is an increasingly accepted collection of techniques to adjust for non-random selection among groups within observational studies. This approach is designed to remove most of the imbalance associated with the *measured *characteristics of cases allowing less biased estimates of treatment effect and other quantities linked with treatment (e.g., cost, quality of life). Propensity scores are designed to extract all of the relevant information from potential confounding variables. A consequence of this method is that two people in the same propensity stratum would be equally likely to receive the (CAM) treatment, after conditioning on all their known pre-treatment characteristics, which justifies an entirely conventional analysis. We will follow the modern literature on propensity scoring to use multiple models with different forms and assumptions, as this is the only way known to probe the validity of the resulting analyses. Table 3 includes a list of EMR variables available to us for this purpose.

**Table 3:** Phase 1: Variables available from EMR for Clustering/Matching Patients

Types/duration of pain condition(s)
Pain severity
Comorbid conditions (e.g., depression, obesity)
Demographics (age, gender, race, marital status, employment status)
Receipt of pain-related disability compensation
Overall healthcare service use
Prescription medications received (e.g., short and long-acting opiates, sedatives, hypnotics, and antidepressants)
Pain-related procedures received (injections, imaging, and surgeries)
Pain-related visits (primary and specialty care including physical therapy)
ER/urgent care visits
Prior use of health insurance reimbursed CAM
Characteristics of primary care provider (e.g., degree [MD, DO, NP] frequency of CAM referrals, gender)

### Analysis of Matched Comparison Groups (MCG)

The third and primary approach we anticipate using to matching participants receiving A/C to suitable controls is the analysis of matched comparison groups. This approach is based on the notion that if, at a particular time, one can form a group of patients who are well-matched on prognostic factors, then within such a group decisions whether to use A/C cannot be based on the matching factors and must be weakly associated with unmeasured factors that are correlated with measured matching factors, thus tending to remove indication bias. If this type of matching were perfectly successful, then the absence of formal randomization would become irrelevant. In the analysis, one would compute treatment effects at the MCG level. Subsequent analysis would search for consistent results across similar MCGs, and in the presence of such similarity, accumulation of results would be applied to patient subpopulations. Formation of MCGs will be aided by the affinity clustering technique [54,67], which can be employed in large samples and has the advantage of identifying an "exemplar" patient whose variables represent those of the MCG members, thus making it possible to characterize MCGs at the analysis stage. Variables from the EMR include the patient characteristics and care variables delineated in Table 3. Available variables include many of those posited to be important factors in patients' decisions to use CAM therapies, as delineated in Figure 1 (Boxes A-C). Our large study patient pool study makes it possible to find MCGs of relatively closely matched CMP patients. We anticipate finding a large number of MCGs, each small and highly homogeneous. Outcomes will consist of various utilization measures, both generally and specific to CMP (see Table 4). We do not anticipate statistical significance at the MCG level due to their small size; rather, we intend to aggregate results across MCGs to investigate homogeneity of A/C effects and to relate the magnitude of A/C effects to MCG exemplar characteristics.

**Table 4:** Phase 1: EMR-derived Clinical Outcomes

Overall use of health care services
Medication use (opiates, sedatives, hypnotics, and antidepressants)
Pain-related procedures (injections, imaging, surgery)
Pain-related visits (primary and specialty care)
Emergency room/Urgent care visits
Evidence of sleep problems
Adverse events attributed to conventional, acupuncture, or chiropractic care treatment for chronic musculoskeletal pain
Mood disorder diagnoses (depression, anxiety)

### Outcome analyses

In identifying MCGs, we will use all CMP patient EMR data collected over a 5-year span (2006-2010). Patients must have a long enough duration of consecutive health plan membership to establish CMP eligibility and evaluate outcomes over the following year. To ensure maximum comparability, eligible patients must have clinical data available over a period when the A/C referral patterns were highly comparable. For each analytic approach, for each outcome measure, an A/C effect (comparing A/C and non-A/C patients) will be computed for each informative MCG. Table 4 shows the EMR-derived clinical outcomes that we plan to examine. We will employ conventional between-group estimates of the A/C effect measures. We anticipate, however, that there may be substantial heterogeneity of A/C effects across MCGs, so that it will be important to characterize where A/C has a beneficial effect, no effect, or an adverse effect. We will use both graphical methods as well as clustering of MCGs. We will also use explanatory linear models, usually ordinary regression, but potentially variants, depending on the specific type of outcome.

#### Multiple levels of analysis

In clinical trials, it is appropriate to pre-specify the main analyses; however, in clinical non-intervention research, the reverse is the case. As emphasized in Guo and Fraser [60], because each analytic approach has its own strengths and weaknesses, it is essential to carry out a multiplicity of different analyses and assess their potentially conflicting results in the context of broad knowledge about clinical processes and patient behaviors. This virtually guarantees that reports of research results from non-intervention studies will be more lengthy and complex than their clinical trial counterparts.

### Sample size considerations

Unlike an RCT, at this stage of EMR-based research we do not have simple, prespecified hypotheses to test. Furthermore, in the light of the desirability of carrying out multiple types of analyses with different endpoints and analysis variants, it is not feasible to make standard sample size computations. Instead, study justification rests on the very large pool of eligible patients (122, 896 health plan members with EMR-identified CMP from 2006 to 2010) and analysis relies on precise estimation of potential effects, rather than on statistical decision-making. Moreover, part of the purpose of an EMR-based study is to portray the variability of response patterns at the individual patient level (as opposed to an average result for the entire sample) to the extent that this is observable. Here again, statistical decision-making does not seem to be an optimal approach.

### Survey of A/C use by CMP patients

Our first-step Phase 1 analyses are confined to variables available in the EMR, which we will use to develop a broadly replicable and efficient approach to evaluating A/C impact. However, patients may be using A/C and other CAM therapies in ways not visible within the EMR. Similarly, there may be patient characteristics contributing to the decision to use A/C therapies that are also not readily visible in the EMR. To address these issues, we will identify patients whose EMR records suggest the presence of CMP within the past two years; we will then invite them to complete a survey identifying current and prior out-of-plan A/C use and use of related treatments/practices. A copy of the survey is available from the first author. We will then replicate the Phase 1 procedures (described above) with the enhanced data set including only those individuals who responded to the survey. This will allow us to evaluate the robustness of our findings by comparing the MCG characteristics identified using only EMR data with those identified in this augmented data set. Additional insights and characteristics predictive of out-of-plan use will help guide sampling of CMP participants for the focus groups in Phase 2, as well as the MCG design for matching participants in Phase 3. Data on out-of-plan use will be most accurate for current use patterns, which are most important for Phases 2 and 3. There will still be "noise" in this data for two reasons: first, not all CMP patients will respond to the survey; second, retrospective self-reporting may not allow us to determine with certainty whether self-care practices or out-of-plan CAM use preceded receipt of covered A/C care. Nonetheless, reporting such practices indicates a patient's general proclivity to seek CAM services or engage in self-care practices to address health concerns. Information on this tendency may enhance our interpretation of patient decision-making and outcomes.

#### Phase 2: Describing the CAM Decision-Making Process Using Qualitative Methods

The second phase of the project will use qualitative research methods to achieve three goals: first, to identify characteristics of A/C received by CMP patients through A/C provider interviews; second, to explore patients' and allopathic providers' decision-making processes in choosing to use/recommend A/C; and third, to identify important outcomes and factors associated with patient and provider satisfaction. Each of these components will inform the construction of instruments for Phase 3.

### Study procedures

For this study phase, we will use qualitative methods because they can elicit the participants' perspectives in defining the range and variability of beliefs, behaviors, and experiences, all within the context of the natural language people use to discuss these issues. Qualitative methods will also allow us to discover themes not anticipated by study investigators. Standardized approaches [68-70] will guide research activities. Our data collection methods will include focus group interviews [71-74] and in-depth interviews [68,75,76], analyzed using a content analysis approach [68].

Through a descriptive case-study approach, we will investigate the full range of details of A/C use, including processes by which allopathic providers decide to make A/C referrals, how CMP patients decide to use such services, and how A/C providers decide the components of care to deliver. The primary questions that guide this case-study research are: (1) What are the barriers and facilitators to seeking/referring A/C for CMP patients? (2) What are the nature and extent of the services received from acupuncturists and chiropractors (i.e., services broader than needling for acupuncture and spinal manipulation for chiropractic care)? and (3) What are the clinical, functional, and quality-of-life outcomes patients and providers associate with receipt of A/C, and which processes or outcomes are most closely associated with patient and/or provider satisfaction?

Phase 2 qualitative data will be collected in two waves: we will use information from the first wave to refine the questions asked during the second wave interviews and focus groups and, ultimately, to collect data on potential confounders (i.e., patient factors related to decisions to use A/C services not reflected in EMR data) for use in Phase 4 analyses.

### Focus groups with patient participants

The following types of patients will be represented among those we interview: (1) patients who were clinician-referred and received A/C services; (2) patients who received A/C without a clinician referral; and (3) comparable CMP patients who have not received A/C (including those referred who did not follow through). We will use data collected through Phase 1 EMR analyses and out-of-plan surveys to identify these types of patients for sampling. We anticipate holding multiple focus groups for each category of participants. Those who received acupuncture and chiropractic care will be recruited for separate focus groups.

Focus groups are an efficient mechanism for investigating how people conceptualize, experience, and talk about issues, for examining a range or consensus of experiences, and for collecting qualitative data from many individuals in a short time. Focus groups will be audio-recorded and consist of 6-12 participants, an optimal size range for discussing behaviors and experiences [73]. Each patient focus group will address the three main topics described above. As part of that process, we will explore two additional issues related to the decision-making process as shown in the model in Figure 1: factors that prompt individuals to seek and, once initiated, continue or discontinue A/C care, and reasons for deciding to seek care with or without primary care provider concurrence. The first wave focus group results will be used to refine the draft Phase 3 questionnaires. Second wave focus groups of prior A/C users will be asked to complete the draft questionnaires prior to focus group attendance and be prepared to advise researchers on domains or questions relevant to their use of A/C services not covered in the questionnaire, as well as to comment on their understanding of the questions we designed. We will not alter questions that are part of validated instruments, but we will use the feedback to refine our overall assessment battery and to adjust items that we have created based on first-wave focus groups.

### Health care provider participants

In in-depth interviews, researchers will introduce a series of prepared, open-ended questions designed to elicit factual information on treatment (A/C providers) and referral (allopathic providers) practices, as well as providers' knowledge, beliefs, attitudes, and experiences relating to A/C for CMP conditions, including what they would perceive to be clinically meaningful improvement. An interview guide, ongoing QA meetings, and transcript reviews will assure uniformity of approach and coverage in the interviews. We will conduct in-depth interviews with acupuncturists and chiropractors who see a high volume of CMP patients from the health plan to learn about their treatment styles and decision-making processes for CMP patients as well as their experiences with KPNW members who are physician-referred or self-referred. We will also interview health plan providers, sampling from among those with high numbers of A/C referrals, those with some A/C referral history, and those who never or almost never refer CMP patients for acupuncture or chiropractic services.

### Qualitative data analysis

Audio recordings of all focus groups and interviews will be transcribed verbatim using standardized transcription protocols. Using content analysis techniques, we will analyze transcripts and field notes [68,77,78] in two stages. First, we will use topical indexing to identify text pertaining to each interview question and prompt, followed by development of a more detailed coding scheme to capture content, themes, and sentiments. Data management and reduction will be supported by use of ATLAS text analysis software (Sage-Scolari, Thousand Oaks, CA). We will compare participant responses within and across categories to identify beliefs, attitudes, behaviors, and experiences important to understanding decisions to seek and continue A/C care and outcomes associated with A/C care for CMP. We will analyze response frequency and content and the vocabulary describing concepts and experiences. This will allow identification of key issues by exploring areas of consensus and contradiction within and across focus group participants and provider respondents [68,79]. Potential bias will be minimized and data credibility enhanced through comprehensive training of interviewers and coders, and multiple members of the research team will complete each type of task. Coder reliability will be determined through check-coding of 1 out of every 6 interviews and focus groups. Because ATLAS can interface with statistical programs, participant characteristics and responses to quantitative questions (e.g., questionnaire draft) can be included in the database and will enable us to retrieve text specific to respondent subgroups.

#### Phase 3: Prospective Cohort Study to Evaluate Outcomes Associated with the Use of A/C Services

To conduct a prospective cohort study of carefully matched A/C and non-A/C patients, we will use clustering and matching methods identified in Phase 1. These matching approaches will be refined by Phase 2 qualitative findings and augmented by additional, directly assessed, patient-reported baseline data. We will follow matched patients to examine outcomes, which will include EMR (service utilization and health care costs) and patient-reported clinical, psychosocial, and QOL outcomes over a one-year prospective timeframe for each patient. We will compare outcomes from the prospective cohort to those found during Phase 1 EMR-only analyses. Specifically, we will conduct the following activities:

1. Set up a weekly surveillance tracking system to identify CMP patients referred for A/C care to allow timely recruitment of these (index) patients and two carefully matched CMP control patients.

2. Coordinate telephone or online assessments for index and comparison patients upon recruitment (baseline; prior to A/C care) and at 1-, 3-, 6-, and 12-month follow-ups.

3. Compare outcomes (clinical and psychosocial/QOL/satisfaction outcomes and health care utilization) and explore health care costs between those receiving A/C and comparison patients not receiving such services, examining relationships between patient characteristics and decisions to use A/C.

4. Conduct comparative and sensitivity analyses between results using methods that rely solely on EMR-derived data (Phase 1) with those supplemented by patient and provider data (Phase 3).

### Phase 3 participants and recruitment

***Index ***participants (i.e., those with an incident A/C referral, n = 200 acupuncture and 200 chiropractic) and matched ***comparison ***participants in this prospective portion of the study must meet the overall CMP eligibility criteria listed in Table 1 and be able to read and respond to assessment questionnaires. Both index and comparison patients must have had no A/C treatment over the previous six months to ensure that we are prospectively following a new episode of A/C care. We purposefully minimized exclusion criteria to ensure that study findings are as broadly representative of the CMP population as possible.

Health plan members will be recruited over a two-year period through a weekly EMR review to identify CMP patients referred for A/C. Those referred for A/C will be mailed an invitation to the study, including a brochure and letter describing the study, and indicating that a staff member will call soon to tell them more about the study. There will also be a study website where prospective participants can log on to learn more about the study and eligibility or opt out if they so choose. Eligible participants will be guided through the consent process and baseline assessments by an interviewer or online, according to their preferences.

### Identification of comparison participants

To recruit comparison cases for each participant in our cohort of A/C users, we will identify CMP-patient matches using the clustering/matching approach determined from Phase 1 and refined based on Phase 2 findings. As with Phase 1, participants must have a primary care visit within one month of the index patients' A/C referral so that we know that an opportunity for A/C referral existed. When an index patient enrolls online, the matching process is triggered: those matches will be located, mailed the same informational materials, and invited to enroll online. We will recruit two or more comparison participants for every index participant. A greater number of comparison participants allows a buffer of comparison participants in the event that some report A/C use during the assessment window.

### Outcome measures and study instruments

The assessment schedule for standardized questionnaires and delineation of EMR-collected outcomes are shown in Table 5. Patient-reported outcomes are measured in a time frame (1-12 months) consistent with many RCTs and other outcome studies of A/C [19,80,81]. Assessment instruments and domains are consistent with the Initiative on Methods, Measurement, and Pain Assessment in Clinical Trials (IMMPACT) recommendations [82,83]. In addition, we include variables representing patient factors influencing the decision to use CAM [43] (Figure 1 plus others identified in Phase 2) to help refine matches of those who do and do not seek A/C.

**Table 5:** Phase 3 Assessment Schedule

	Assessment Month				
	0	1	3	6	12
**Chronic Musculoskeletal Pain Patient Measures:**					
**Pain and related disability (patient-reported, primary outcomes)**					
Pain Severity (BPI-SF subscale)	√	√	√	√	√
Pain Interference (BPI-SF subscale)	√	√	√	√	√
Pain Bothersomeness (single item)	√	√	√	√	√
**Secondary Outcomes (survey instruments)**					
Overall well-being (Arizona Integrative Outcomes Scale)	√	√	√	√	√
Patient global impression of change		√	√	√	√
Quality of sleep (ISI)	√	√	√	√	√
Work- and activity-related impairment (NHIS questions)	√	√	√	√	√
Depression (PHQ-8)^1^	√	√	√	√	√
Anxiety (GAD-2)^1^	√	√	√	√	√
Quality of Life/health utility index (SF-12)	√	√	√	√	√
Patient satisfaction		√	√		
**Secondary outcomes (health care utilization collected through EMR and administrative records)**					
Overall use of healthcare services and associated costs		EMR^2^	EMR	EMR	EMR
Use of medications (short and long-acting opiates, sedatives, hypnotics, antidepressants)		EMR	EMR	EMR	EMR
Pain-related procedures (injections and imaging)		EMR	EMR	EMR	EMR
Pain-related visits (primary and specialty care)		EMR	EMR	EMR	EMR
ER/urgent care visits		EMR	EMR	EMR	EMR
Adverse events associated with conventional and CAM CMP treatment^3^		EMR	EMR	EMR	EMR
Use of health insurance reimbursed CAM		A^4^	A	A	A
**Patient characteristics and potential moderators (including predisposing, enabling, and need factors associated with A/C use decision from **Figure 1)					
Demographics	√				
Received/seeking pain-related disability compensation	√				
Patient expectations/CAM attitudes	√				
Other characteristics of pain condition (type(s)/duration)	√				
Previous use of acupuncture/chiropractic services (adapted from NHIS survey)	√				
Utilization of non-plan CAM health services & products	√		√	√	√
**Healthcare Provider (Allopaths/Acupuncturists/Chiropractors) Measures:**					
Allopathic provider characteristics and CAM beliefs	√				
Acupuncturist/chiropractic service provided & general practices			√	√	

Note: ^1^depression and anxiety about pain will also be examined as potential moderators of pain outcomes; ^2^EMR = electronic medical record, ^3^participants will also be asked directly about CMP treatment-related adverse events; ^4^A = administrative record;

#### Primary outcomes: Pain and related disability

Two subscales--the 4-item pain severity and the 7-item pain interference subscales--from the short form of the Brief Pain Inventory (BPI-SF)[84,85] will be used to assess pain and related disability. The BPI has sound psychometrics and has been widely adopted for clinical pain assessment, epidemiological studies, and studies of treatment efficacy. We will also include a measure of how bothered participants are by their pain. This instrument uses a 0 to 10 scale of "symptom bothersomeness, " where 0 represents "not at all bothersome" and 10 is "extremely bothersome." This question has been frequently used in studies of back pain [18,19,86] and shown to have adequate construct validity [87].

#### Secondary outcomes (patient-reported)

a. Global assessment of change. The Patient Global Impression of Change scale (PGIC [88]) will be used to evaluate participants' overall evaluation of the impact of their A/C treatment.

b. Work-related impairment. Three questions about work/disability status and missed work due to CMP will be adapted from the National Health Information Survey (NHIS) [89].

c. Depression severity. Using the Personal Health Questionnaire (PHQ-8) [90,91], we will measure depression severity. The PHQ-8 is established as a valid screener and severity measure for depressive disorders in large clinical studies [90,92-94]. Baseline depression will also be examined as a possible moderator of outcome.

d. Anxiety. The 2-item Generalized Anxiety Disorder Scale (GAD-2) [95,96] will be used to screen for anxiety disorders. The GAD-2 has been used to detect anxiety disorders in primary care and has well established psychometric properties. Baseline GAD-2 scores will also be examined as another possible moderator of outcome.

e. Quality of life/health utility. The SF Health Survey (SF-12v2) [97] will be used to measure quality of life. The SF12 is a widely used and well-validated generic measure of functioning that is a recommended IMMPACT measure for examining functioning (this will also be used as a health utility measure) [82].

f. Patient satisfaction. Finally, participant satisfaction with services provided by the health plan (allopathic providers) (0 = worst health care possible, 10 = best health care possible) will be assessed as well as satisfaction with A/C provider when pertinent [98]. We will also use the practitioner skill subscale from a self-report outcome measure designed for assessing CAM treatments [99,100].

#### Secondary outcomes (health care utilization through EMR and administrative records)

These EMR-based outcome measures were listed in Table 4 as they also form the basis for our Phase 1 outcome analyses.

*Patient characteristics and potential moderators *include factors that may influence decisions to use CAM (see Figure 1)[101] or that have been found to moderate pain-related outcomes [102-104]. These factors might then be used as additional baseline matching variables or covariates to maximize comparability between index- and matched comparison-participants included in the analyses. Demographics include age, gender, race/ethnicity, education, marital status, employment status, income, and income source. Administrative records will identify participants seeking or receiving worker's compensation or other disability payments for their CMP. This will be augmented by a single-item question about disability status of participants on the questionnaire. We will assess general preventive behavior [105,106] by collecting data on self-management behaviors including physical activity practices. In addition, we will assess participants' general expectations of CMP improvement, improvements with A/C treatment [27,31], and how helpful a variety of CAM services and practices (provider- and self-directed) may be [107]. We will measure other characteristics of the participant's pain condition, including age of onset, perceived etiology of CMP, and type(s) of current CMP. Finally, we will collect data on use of other CAM services/products, including any CAM treatment visits not referred through the health plan (e.g., chiropractic and acupuncture, massage therapists, naturopaths) as well as CAM-related self-care practices (e.g., yoga, meditation, tai chi) and products (e.g., dietary supplements).

#### Health care provider measures

a. Allopathic provider characteristics and factors associated with A/C referrals including questions describing general provider characteristics (i.e., gender, specialty type, professional degree, years in practice, and training/expertise in CMP) and A/C referral attitudes, as well as an EMR-based variable reflecting the inclination for a given provider to refer for A/C services (referral rate per CMP patients on panel).

b. A/C provider characteristics and treatment patterns include questions adapted from the A/C-visit data forms developed by Sherman and colleagues [108] and refined during our Phase 2 interviews with the CAM providers. We also have access to data collected by the health plan on CAM-referred services patients receive (e.g., number of sessions; assigned diagnostic, procedure, and treatment [CPT] codes.

### Phase 3 Data Analysis

#### Patient-reported outcomes

For the patient-reported outcomes in Phase 3, we will examine differences in baseline measures between participants with and without data at the 1-, 3-, 6-, and 12-month  follow-ups. Variables related to missingness will be included in the main analyses as covariates to reduce bias in the estimates [109,110].

We will use multilevel modeling to examine differences across time in primary (pain intensity/interference/bothersomeness) and secondary patient-reported outcomes between A/C users and matched comparison participants. Two parameters (linear and quadratic slope) will characterize change across time, with linear slope capturing initial rate of change and quadratic slope reflecting the degree to which the change slowed (or increased) over time. Thus, the quadratic model for time (baseline and 1-, 3-, 6-, and 12-month follow-ups) captures nonlinear change across time. Unlike repeated measures analysis of variance, multilevel modeling does not require data at all time points from each subject, so data from all subjects will be included in analyses. Depending upon the distribution, normal, logistic, or Poisson models will be used. Importantly, these analyses can be adjusted using additional information collected from participants during the baseline assessment that we may identify in Phase 2 as influencing A/C decision-making. This will better control for factors not available during comparison patient identification when only EMR data will be available for matching/clustering.

#### EMR-related outcomes

To examine total health care utilization across 12 months, the analysis issues laid out for Phase 1 continue to apply. Using the MCG as the unit of analysis, the conventional statistical methods from Phase 1 continuous measures will be assessed by one-sample location tests and discrete outcomes will be assessed by binomial methods or by ordinal multinomial methods in the case of multiple outcomes. Like Phase 1, MCG characteristics in the Phase 3 sample will be related to A/C effects using the models described above. Implementing the same analytic strategy here as in Phase 1 (i.e., using EMR data alone), we will be able to test directly for the contribution provided by the additional prospective information on patient-and-provider characteristics (e.g., pain severity, self-care behaviors, provider attitudes to A/C referral). We will do so by including patient-supplied variables at the patient level in the analytic model and then introducing random-effects terms for the MCGs to account for within-MCG correlation.

#### Costs

Health plan costs are one of our secondary outcomes, as we are interested in how total health plan costs differed between A/C users and nonusers. Costs will be estimated by applying internal unit costs developed and tested in previous studies [111,112] to the HMO patient-level utilization measures, with the final cost variables acting as proxies for HMO resource cost. We are interested in the effect of patient-level factors--in particular, patients' use (or not) of A/C services on health care costs. Admittedly, the challenges of analyzing typical cost data are well known and significant; these include 1) having a large proportion of non-users (i.e., zero costs); 2) the fact that non-zero costs are usually right-skewed; and 3) heteroscedasticity. Assuming these challenges exist in our study cost data, we will tackle them by exploring transformation models and generalized linear models (GLM). Transformation models convert skewed cost data using a transformation (e.g., log-normal) to a better-behaved distribution (i.e., one more normal and symmetric, homoscedastic, less skewed, promoting more efficient estimation). A complication of transformations is possible re-transformation bias in switching from the transformed scale to the original dollar-based scale. GLM avoids re-transformation bias but can be highly imprecise if the residual pattern is misspecified. We will explore both model forms to determine the most accurate and descriptive approach for our study data. Cost analyses will be performed in STATA version 11.

### Phase 3 Sample Size

We based our Phase 3 sample size determination on the BPI short form severity (BPI-S) and interference (BPI-I) subscales. Although these outcome measures are highly recommended for pain studies [82], it is difficult to find the appropriate statistics reported in the literature, that are required for design purposes. We have relied on the findings from the SCAMP intervention [113,114] and Tan's and colleagues' validation study of the use of the BPI for chronic nonmalignant pain [115], who reported SD = 2 (approximately) for both the severity and interference subscales of the BPI in large samples of patients with nonmalignant pain. Changes on the order of 1-3 points have been cited as being relatively common [116] and within the range of change found in the SCAMP intervention [113,114]. Collectively, these sources suggest that an attainable and clinically meaningful effect size would be 0.50, which translates into a difference between mean changes of 1 unit (absolute). For 100 patients in each of two groups, assuming a 0.5 correlation between pre- and post-measurements, using a conventional two-sample t-test with a two-sided significance level of 0.025 (to allow for two tests), the power would be slightly over 90% to detect a 1 unit treatment effect. The analysis we intend to use would be more efficient, because it would adjust for baseline BPI values, but we might also want to include explanatory factors into the analysis, which might decrease efficiency. Consequently, we propose group sizes of 200, for a total of 1, 200 - that is a total of 200 participants each for those receiving acupuncture and chiropractic services as well as two matched comparison CMP participants for each of these index participants for a total sample size of 1, 200. Table 6 shows sample and effect sizes for a number of recent studies of related pain conditions utilizing related measures as well as anticipated effect size estimates for the current study. While these comparative clinical trials may have possibly produced greater effect sizes than we will observe in this observational study, our proposed group size is substantially larger than those reported for each of these studies.

**Table 6:** Sample and Effect Sizes for Related and Current Study

Study	Condition	Treatment	Assessment Scale	Group size	Effect size
Cherkin, 2009 [19]	chronic LBP	acu	RMDQ	160	0.46
Cherkin, 2009 [19]	chronic LBP	acu	bothersomeness	160	0.42
Liang, 2011 [118]	chronic neck pain	acu	NPQ	81	0.24
Franca, 2008 [119]	tension headache	acu v PT	VAS pain	16	2.0
Molsberger, 2010 [120]	shoulder pain	acu v UC	VAS pain	150	1.01
Hondras, 2009 [121]	LBP > 55 yrs	chiro v UC	RMDQ	90	0.3
Kroenke, 2009 (as reported in Krebs, 2010)[113,114]	musculoskeletal pain	Rx/self care v UC	BPI-S, BPI-I	125	0.56 (BPI-S) 0.59 (BPI-I)
Current study	musculoskeletal pain	acu v UC	BPI-S & BPI-I	200	0.5*
Current study	musculoskeletal pain	chiro v UC	BPI-S & BPI-I	200	0.5*

Note: LBP - lower back pain; acu - acupuncture; chiro - chiropractic care; LM - light massage; RMDQ - Roland Morris Disability Questionnaire; NPQ - Northwick Park Neck Pain Questionnaire; BPI-S - Brief Pain Inventory Severity Subscale; BPI-I - Brief Pain Inventory Interference Subscale; * estimated ES

**Figure 3:**
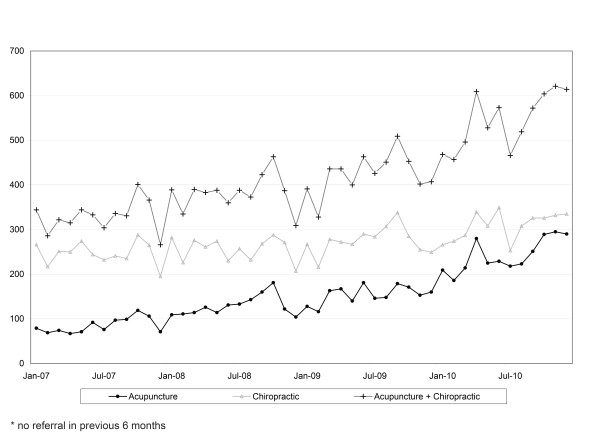
Health Plan Patients with Chronic Musculoskeletal Pain (CMP) Receiving New* Acupuncture and or Chiropractic Care (A/C) Referral.

To estimate available numbers of CMP patients who may receive A/C care for the Phase 3 prospective cohort study, we examined the number of health plan referrals for A/C care during a recent three-year period. Referral patterns for A/C among health plan members with CMP (see Figure 3) are fairly stable, with an average of 330 acupuncture and 303 chiropractic referrals monthly; therefore, we should have an adequate recruitment pool (estimate of 15, 192 A/C referrals with CMP over the two-year recruitment period) to meet the target enrollment for this phase of the study.

### Ethical Approval

Written consent will be obtained from each participant in Phases 2 and 3 and from those completing the out-of-plan A/C survey in Phase 1. For the data-only portion of Phase 1, health plan members' contracts with the HMO provide consent for use of their data in HMO-sponsored research studies. This study protocol was approved by the institutional review boards (IRB) of Kaiser Permanente Northwest, University of Arizona, and Oregon Health and Science University. This study protocol is registered with http://www.clinicaltrials.gov (NCT01345409).

## Discussion

We expect that successful completion of this study will provide an evaluation of the outcomes--from the perspectives of patients, providers, and health services utilization--associated with the real-world use of A/C services. By comparing the results of the EMR-only analyses with the more comprehensive results available in Phase 3, we will be able to identify the strengths and weaknesses of EMR-alone analyses and potentially identify the most critical additional data needed. We further anticipate that the trio of EMR-based analyses, qualitative assessment, and prospective cohort studies will provide a clearer understanding of the decision-making processes behind the use of A/C for CMP; these will also provide a transportable methodology that can be applied to other health care settings, CAM treatments, and clinical populations.

With regard to advancing our understanding of patients' use of CAM in everyday practice settings, much CAM research to date has focused narrowly on "early adopters" (those likely to use CAM services regardless of barriers [117]). Yet within our cohort, cost and access barriers are substantially reduced, allowing us to study other factors (see Figure 1) influencing A/C care receipt and outcomes and to develop a comprehensive characterization of A/C use patterns and outcomes in patients with increasing access to such CAM services. An important goal of this study is to clarify how CMP patients use A/C compared to conventional care (to augment or replace it), when and why patients disclose A/C use to allopathic providers, and how such practices may vary by patient characteristics. Our mixed methods approach will help us develop a better understanding of the process behind the decision to initiate (patients), recommend (allopathic providers), and continue (patients and providers) A/C care for the treatment of CMP. Finally, studying this in everyday treatment settings allows us to consider the range of services received in A/C care (e.g., needling or spinal manipulation vs. whole systems-based care) and variation by characteristics of the CMP patient and/or A/C provider.

In addition to advancing our understanding of real world A/C practices and their impact on CMP, this study affords us the opportunity to build upon the increasingly available EMR systems to conduct research that may be more broadly adopted to better understand the real world interplay of conventional medicine and A/C services. Using EMRs exclusively may limit information on important variables (covariates) on which to match or cluster like patients. However, by augmenting data sets through direct reports by providers and patients regarding important decision-making factors suggested by qualitative findings, we may substantially reduce potential confounding in comparing those who do and do not receive A/C treatment. Thus, we can identify whether widely available data in EMR and administrative systems can be used to adequately forecast patients' outcomes and evaluate the extent to which supplementation from additional patient- and provider-collected information is a necessary adjunct to EMR-based data analysis.

Finally, our MCG-focused methodology gives us a unique opportunity to conduct patient-centered research; that is, to identify like groups based upon actual individual patient care characteristics rather than hypothetical group averages. A typical RCT may not include enough participants to form homogeneous clusters for separate evaluation, yet an EMR database of a large clinical population like the CMP sample described here does include sufficient numbers of patients with similar characteristics and disease severity to form clinically meaningful clusters as the basis of the analyses. Accordingly, this research may allow us to address the clinical question as follows: for whom does supplementary CAM care result in clinically significant improvements in pain severity and overall functioning? Our planned MCG methodology will allow us to identify the characteristics of CMP patients for whom A/C may have the greatest impact on satisfaction, functioning, clinical, and QOL outcomes; it will also clarify whether the timing of services relative to conventional care and the course of the CMP affects such outcomes.

## List of abbreviations

CAM: complementary and alternative medicine; A/C: acupuncture/chiropractic care; ACU: acupuncture; CHIRO: chiropractic; LM: light massage; CP: chronic pain; CMP: chronic musculoskeletal pain; TMD: temporomandibular disorders; HMO: health maintenance organization; KPNW: Kaiser Permanente Northwest; EMR: electronic medical record; AR: administrative record; ICD-9: International Statistical Classification of Diseases - 9^th ^edition codes; CPT: current procedural terminology codes; MCG: matched comparison groups; SD: standard deviation; ES: effect size; GLM: general linear models; RCT: randomized clinical trial; QA: quality assurance; MD: medical doctor; DO: doctor of osteopathic medicine; NP: nurse practitioner; IMMPACT: Initiative on Methods, Measurement, and Pain Assessment in Clinical Trials; BPI-SF: Brief Pain Inventory-Short Form; BPI-S: Brief Pain Inventory Severity Subscale; BPI-I: Brief Pain Inventory Interference Subscale; PGIC: Patient Global Impression of Change; NHIS: National Health Information Survey; PHQ-8: Personal Health Questionnaire-8 item version; GAD-2: Generalized Anxiety Disorder-2 item version; RMDQ: Roland Morris Disability Questionnaire; NPQ: Northwick Park Neck Pain Questionnaire

## Competing interests

The authors declare that they have no competing interests.

## Authors' contributions

All authors contributed to the design of this study protocol. All authors read and approved the final manuscript.

## Pre-publication history

The pre-publication history for this paper can be accessed here:

http://www.biomedcentral.com/1472-6882/11/118/prepub
